# Loneliness, coping practices and masculinities in later life: Findings from a study of older men living alone in England

**DOI:** 10.1111/hsc.13732

**Published:** 2022-01-26

**Authors:** Paul Willis, Alex Vickery

**Affiliations:** ^1^ 1980 Associate Professor in Social Work and Social Gerontology School for Policy Studies University of Bristol Bristol UK; ^2^ Senior Research Associate School for Policy Studies University of Bristol Bristol UK

**Keywords:** ageing, community‐based interventions, groups, loneliness, old age, older men, social isolation

## Abstract

While much attention has been given to loneliness as a public health and societal problem less consideration has been given to men's experiences, particularly in later life, and there is a limited evidence based on what works with supporting older men to counteract the impact of loneliness on their mental and social wellbeing. In this paper, we focus on the experiences of older men living alone in the community. Between 2017–2018 72 men residing in England (65–95 years) took part in the study and shared their accounts of experiencing and addressing loneliness on an everyday basis. We generated qualitative data through semi‐structured interviews. Interview data were analysed thematically using the framework analysis approach. We present and discuss findings on the difficulties older men experience in responding to and discussing loneliness and the range of coping practices men applied within their home environments. Three core themes are presented: 1. Maintaining silence around loneliness and distress; 2. Getting on with it versus feeling stuck in loneliness and, 3. Temporary remedies and distractions from loneliness from within the home. Across men's accounts, ‘the home’ is constructed as a space of biographical connections with others as well as a restrictive environment of separation from others. In relation to help‐seeking and efforts to break silence surrounding loneliness, the findings speak to the ways in which masculinities and social expectations attached to male roles complicate older men's varying attempts at help‐seeking and underpin some of their everyday methods of coping with loneliness. The findings reiterate the importance of targeted individual support for older men, particularly for those feeling frozen in loneliness. Facilitators of group‐based support for older men need to recognise the different and potentially conflicting positions older male service users may adopt in relation to help‐seeking that are configured around masculine ideals but in diverging ways.

**What is known about this topic?**

- Older adults make up the largest group living alone. Living alone is an identified risk factor for loneliness;
- Not much attention has been given to older men's experiences of loneliness and their coping practices;
- Masculinities complicate men's attempts to seek help with physical and mental health in later life.

**What this paper adds?**

- Findings highlight how older men cope with loneliness on an everyday basis in and around the home;
- Within the findings the home is represented as a restrictive environment (compounding loneliness) and a space of biographical connection (bringing comfort in solitude);
- Hegemonic masculinities shape older men's attempts to speak about loneliness and can be experienced as enabling *and* immobilising in addressing loneliness.

## 1 INTRODUCTION

The focus of this paper is on older men's experiences of responding to and coping alone with feelings of loneliness. We attend to the unique challenges older men who are living alone experience in discussing loneliness with significant others and the coping practices they rely on to alleviate feelings of loneliness within and external to the home. We focus on this population for two reasons. First, an increasing number of adults are living alone in the United Kingdom (UK), of which older adults make up the largest number of ‘one‐person households’ (ONS, 2019). Second, men's experiences, encompassing the intersection between masculinities and older age, are rarely examined separately in current approaches to understanding and addressing loneliness. Men in later life experience unique challenges in maintaining social connections following key turning points such as transition to retirement, widowhood or separation and divorce (Dykstra & Fokkema, 2007; Hearn, 1995).

Studies of ‘men‐only’ groups that focus on men's interests and activities suggest these gendered spaces are more appealing to men, for example, research on Men's Sheds internationally including the UK, Australia, Ireland and Canada (Milligan et al., 2015; Nurmi et al., 2018; Wilson & Cordier, 2013). Other authors question the premise of these spaces as more appealing and suggest this may operate as a barrier for some men (Ratcliffe et al., 2021). What is missing more broadly is a focus on how older men respond to and cope with loneliness on an individual level separate from group‐based interventions.

Our central aim is to examine the everyday coping practices older men exercise within the private sphere of the home. An overarching objective is to enhance current understanding of how older men living alone respond to and cope with loneliness solo. We report qualitative findings from an English study of community‐dwelling men (65–95 years of age) and their social connections. Across men's accounts, ‘the home’ was constructed as a space of social connection *and* separation from others. The findings speak to the ways in which masculinities and social expectations attached to traditional male roles, coupled with the stigma associated with loneliness, complicate older men's varying attempts at help‐seeking and underpin some of their everyday methods of coping with loneliness.

## 2 BACKGROUND LITERATURE: LONELINESS AND COPING PRACTICES

Loneliness is typified as a negative emotional state that arises from a perceived discrepancy between the relationships we have and the ones we desire (De Jong‐Gierveld, 1987; Perlman & Peplau, 1981). Loneliness is a relational state that hinges on an individual's subjective awareness of what it means to experience meaningful and rewarding relationships with others (Jylhä & Saarenheimo, 2010). Across the literature on loneliness three types stand out: emotional (Weiss, 1973); social (De Jong Gierveld & Tilburg, 2006; Perlman & Peplau, 1981; Weiss, 1973); and, existential (Bolmsjö, Tengland & Rämgård, 2018; Ettema, Derksen & van Leeuwen, 2010). While overlapping, loneliness, living alone and social isolation are distinguishable concepts and living alone is not a reliable measure of loneliness (Smith & Victor, 2019). This troubles the assumed associations between older age, social isolation and loneliness (Barreto et al., 2021) and invites a rethink of older people's capacity to maintain active social networks in later life. In contrast to loneliness, social isolation infers an absence of contact with others. Living alone is one criterion by which it is measured (Beach & Bamford, 2013; Gale et al., 2018). Periods of isolation can generate subjective states of solitude or loneliness; the former being characterised as constructive while the latter recognised as disruptive to one's social and emotional wellbeing (Ettema et al., 2010).

The ways in which older adults cope with loneliness have been examined in tandem with studies of loneliness. Categories of coping typify common ways in which people respond to stress (Skinner et al., 2003). In this context, we are interested in the emotional stress generated by feelings of loneliness. Schoenmakers et al. (2012; 2015) identify two pathways for coping with loneliness – problem‐based by seeking to improve relationships with others, and emotion‐focused by seeking to change one's expectations about relationships. Their survey findings suggest older adults practice both pathways, however, individuals in higher age groups look more to emotional regulation strategies (i.e., lowering expectations) than means of improving relationships (Schoenmakers et al., 2012). The degrees to which older adults experience loneliness influences pathway preferences with those who have been persistently lonely for longer more likely to lower their expectations of others (Schoenmakers et al., 2015). Morgan and Burholt (2020) contend that loneliness represents a source of biographical disruption that negatively impacts an individual's sense of self and self‐worth. Equally, older people adapt to this disruption by reconstructing their perceptions of self‐identity. One way of alleviating loneliness and improving social connections is through group‐based participation. Published reviews on group‐based interventions indicate some positive outcomes, such as reductions in reports of loneliness, with scope for increasing the evidence base on what works well in implementing group‐based programmes (Cattan et al., 2005; Gardiner et al., 2018; Hagan et al., 2014).

Another stream of literature that resonates with the focus of this study is scholarship on the intersections between masculinities, health, and help‐seeking. In the context of health and illness, a prominent theme is the relationship between maintaining masculinity and exhibiting ‘traditional masculine beliefs’ for men and delays in help‐seeking behaviours (Addis & Mahalik, 2003; Courtenay, 2000; Galdas et al., 2005; O’Brien et al., 2005). Connell (1995) refers to the most culturally dominant masculinity at a given time as hegemonic masculinity, defining successful ways of being a man in particular places at specific times. Hegemonic masculine practices depict strength, stoicism, self‐reliance and autonomy, as well as dominance and superiority over others. Men construct hegemonic masculinity through dismissing health care needs and health promoting behaviours (Courtenay, 2000). Tannenbaum and Frank (2011) argue that the desire to uphold hegemonic masculine ideals of independence, self‐reliance and dismissal of pain and illness is embedded in older men's health beliefs and behaviours. Other research on help‐seeking indicates similar themes with older men delaying support‐seeking until a crisis point arises (McVittie & Willock, 2006) and preferring to act independent of others when making decisions about their health and wellbeing (Smith et al., 2007).

It is, however, important to consider how hegemonic masculinity as an explanatory construct is not always universally accepted. Other work challenges the notion that gendered power can explain social disadvantage and men's behaviours and experiences. Holter (2014) for example, proposes an explanatory model that includes structural factors as well as masculinity changes, highlighting how there are changing hegemonic masculinity forms which allow for more equality between men and women, as well as between men. Thus, we need to consider societal and structural factors, together with variation amongst men (Holter, 2014). In this paper we examine older men's responses to feelings of loneliness and their everyday strategies for coping in the context of living alone.

## 3 METHODS

### 3.1 Study design

Themes presented below are from a cross‐sectional qualitative study on older men's experiences of loneliness and social connections in later life in England (2016–19). One hundred and eleven men (65–95 years) took part. Men were purposively recruited to five groups: (1) men who were single or living in urban areas (*n* = 21); (2) men who are single or living alone in rural areas (*n* = 22); (3) men who identified as gay and were single or living alone (*n* = 21); (4) men who were carers for significant others (*n* = 25); and (5) men with hearing loss (*n* = 21). Participants were recruited through groups and services for older people, particularly older adults belonging to these five groups. A recruitment list of public, third sector and voluntary organisations, as well as social clubs and societies, was drafted. Stakeholders from each site were asked to advertise the study to older men accessing their programme or service.

All men took part in a single semi‐structured interview, running between 1.5 to 2 hr duration. Most elected to be interviewed at home. The interview included completion of a visual social network exercise to explore the membership composition of men's current social networks and recent changes to their network membership. Other questions explored participants’ social background, including work‐life; experiences of loneliness; ways of coping; and, participation in local groups and societies. See Table 1 for a fuller list of types of questions asked and sample questions.

**TABLE 1:** Interview schedule, question categories and sample questions

Question categories (in sequence asked)	Sample questions asked
1. Introduction and participant background	Can you tell me a bit about where you grew up? How long have you lived in this area? (*follow up*) What brought you here? What kinds of jobs have you had through your life? What hobbies and interests have you enjoyed doing in your life? Any voluntary activities? What about current hobbies /interests/ activities? Do you have any pets? If yes: how important are they to you?
2. Social networks exercise^b^	Who do you spend most time with? What ways do you have of keeping in touch with them? (e.g. face to face, phone, text, email, Facebook) Are there any things that make it difficult to keep in contact with any of them? If so, what? Is there anything you would like to change about your contact with them? If so, what (type of contact, frequency, location, quality) Have there been any changes to your network over the last ten years? If so, what (grown, got smaller, circumstances, feelings) Who lives in the same household as you? And who lives in the immediate neighbourhood but not same household? And apart from these, who lives within 1 hr travelling distance? Who do you consider to be friends? Who do you consider to be family? Who would you confide in about personal problems or worries? Who would you approach for practice help and support?
3. Experiences of loneliness	How would you describe loneliness? How can it make other people feel? Have there been times when you have had similar feelings? If so, what did this look like? Can you tell me about what was happening in your life at those times? Are there particular times or situations when you are more likely to feel lonely? Has loneliness ever got to the point of becoming a problem in your life? (when? what happened?, how long did it last?) In terms of how often you have feelings associated with being lonely^a^, *Do you ever feel that you lack companionship?* If so, how often? What situations prompt this? *Do you ever feel left out?* If so, how often? What circumstances prompt this? *Do you ever feel isolated from others?* If so, how often? What circumstances prompt this? (e.g. your own / partner's health, finances, separation / bereavement)
4. Coping with loneliness	When loneliness has been a problem in your life, what kinds of things have you found that help? What helps to combat loneliness or to feel less lonely? How? Who has been helpful in tackling loneliness? What makes them helpful? Who do you look forward to seeing and talking to day‐to‐day? What do you appreciate about their company? How do you connect with them (e.g. face to face, online / social media)
5. Participation in group activities	Thinking about all the groups and activities you take part in, what are they and what are they like? Where are they? What goes on there? How did you first hear about them? What were your views about them before you went there? What do you like about taking part? Who are the other people that take part? What things do you get out of taking part? Have there been any challenges about taking part? If so, what? (e.g. transport, finance, technology, disability) How do these groups compare with the contact with the people in the social convoy mapping exercise? Is there anything that has prevented you from taking part in a group that you would have wanted to take part in? If so, what? Are there other things you would like to do in a group, but which don't exist? What other people would you like to have in the group? What's the best thing about being involved in …. [group]? How would you recommend it to others?

^a^ During interviews, we asked each participant to complete a social convoy mapping exercise to understand their social networks and what supports enable them to cope with stressful life‐situations and challenges (Antonucci, 2001). This illustrative method uses a diagram of three concentric circles to identify and explore the personal and situational characteristics of an individual's social network membership, and how important they are to them, both at the time of interview and ten years earlier (Antonucci, 1986). The sample questions above are about network members listed by participants.

^b^ These three questions (*Do you ever feel that you lack companionship? Do you ever feel left out? Do you ever feel isolated from others?*) were taken from the UCLA three‐item loneliness scale (Hughes, Waite, Hawkley, and Cacioppo, 2004). While not intended for qualitative research, these open‐ended questions were appealing as they invited participants to explore dimensions related to subjective feelings of loneliness, and were most beneficial when participants did not want to discuss loneliness or gave very brief responses to other loneliness questions.

The study received ethical approval from the NHS Social Care Research Ethics Committee (REC ref 17/IEC08/0004). Participants were provided with an information sheet outlining study involvement and data management and provided written consent prior to interview, including consent for the audio recording of interviews. All participants were provided with a list of local support services and groups targeted at men and older adults more generally at the end of interviews.

### 3.2 Sample characteristics

Men taking part were between 65–95 years of age (mean age 76). Majority of participants were from White British backgrounds with six men identifying with the following ethnic groups: Pakistani, Asian, South African, Indian and Black Caribbean. For the purpose of this paper, we examined data from a sub‐sample of 72 men who were living alone at the time of participation—see Table 2. Twenty‐one identified as gay; other men identified as heterosexual.

**TABLE 2:** Characteristics of participants living alone

Total number living alone	72
Age range	65–95
Mean age	76
Ethnic background	White British = 68 Asian = 4
Sexual identity	Heterosexual = 51 Gay^a^ = 21

^a^ Two gay men lived alone with their partners living in another household. One man was single but sharing his house with a part‐time lodger, therefore we included him in this sub‐sample.

### 3.3 Analysis

Interview transcripts were uploaded to NVIVO11 for data management and analysis. Data were categorised across a matrix, following the framework approach for qualitative data management (Gale et al., 2013). To develop the coding framework, three team members read a sample of transcripts that incorporated priori categories identified in the literature, topics from the interview schedule, and categories arising inductively. After confirmation of categories, the framework was populated with data charted from the transcripts. Categorical data summaries were thematically analysed using an iterative process of moving between initial coding line‐by‐line and defining and naming recurrent themes across the dataset (Braun & Clarke, 2006). Across summaries, core themes were identified that conveyed ‘stories about particular patterns of shared meaning’ and which were agreed between authors (Braun & Clarke, 2019, p. 592).

## 4 FINDINGS

When reflecting on experiences of loneliness, participants identified major changes and upheavals in their social circumstances and intimate relationships that they associated with subsequent feelings of loneliness. The most common events were: death of a spouse or partner and the overlap between bereavement and loneliness (discussed by over a third of the sample); divorce or separation from long‐term partners; extended periods of illness and poor health that restricted levels of sociability; and, retirement and the associated loss of social bonds formed within workplaces. In other papers, we have discussed how older men experience loneliness across different groups (see Willis et al., 2020, 2022). Here, we concentrate on three core themes: 1. Maintaining silence around loneliness and distress; 2. Getting on with it versus feeling stuck in loneliness and, 3. Temporary remedies and distractions from loneliness.

### 4.1 Maintaining silence around loneliness and distress

Two main barriers were identified that prevented participants from discussing loneliness with others: (1) concerns about the stigma and embarrassment attached to being alone, coupled with the perception that others (friends, family) were not interested in their lives, and (2) avoidance of burdening others (particularly adult children) with their problems:

That’s my feelings, I’ve kept private. Not through a sense of not wanting to discuss things with people, but a feeling that either they’re not interested in you personally, or that you will be embarrassed by talking about intimate things like that [M63, 67, gay]

One participant who described himself as socially active reflected on his reluctance to broach the topic with friends:

I don’t bang on about me being a bit lonely at times because I don’t think they’d really want to hear that anyway. My mates, the ones I play football with, have said, “Oh, if you’re ever lonely, come along,” but what people say and what they do, I’ve found, are totally different things. I’ve always been pretty independent anyway, so I don’t push myself on people. [M4, 65, heterosexual]

His comments indicate a perception that other men do not want to hear about friends experiencing loneliness and are not receptive to these conversations.

Participants who were fathers discussed how feelings of loneliness were not in keeping with their familial role:

That’s a difficult one, that’s a difficult one. Because it certainly wouldn’t be the family, because I feel the family looks on the father as the top, and nothing goes wrong with their father, you know. So, to go to them with a problem, it wouldn’t work. [M13, 82, heterosexual]

Relating to this, there was some participants who did not disclose emotional distress or associated feelings of loneliness to avoid worrying those close to them:

Not talking to my family in all honesty, I don’t talk to my family much about it. They get upset at seeing me upset, so I don’t do it. …I don’t really talk about the situation which is affecting me. [M61, 75, heterosexual]

Heterosexual participants conveyed gendered pressures to maintain expectations of leadership and protection attached to fatherhood roles and to not reveal vulnerabilities to other family members, particularly adult children.

Some participants alluded to interpersonal challenges unique to men that inhibited them from sharing feelings of distress, often characterising as men tending to ‘bottle things up’. Others recounted difficulties in speaking to friends and helping professionals about mental distress:

I could only talk to you [interviewer] because you’re doing research or you’re a bit distanced. I don’t know you at all. Men don’t normally talk. Some are different. My family is very much, “You make your bed and you lie in it.” [M9, 78, heterosexual]

His comments indicate it is easier to speak to a stranger than family, particularly if family members are unsympathetic. He also acknowledged that the trope ‘men don't normally talk’ does not apply to all men, and there are other differences at play.

### 4.2 Getting on with it versus feeling stuck in loneliness

Across men's accounts, comments on help‐seeking fluctuated between two contrasting positions. Some men described being stuck in a state of loneliness and conveyed a sense of social inertia (i.e., ‘I don't know what to do about it’). Others emphasised self‐sufficiency and self‐reliance in solving the problem of loneliness and reaching out to others (i.e., ‘doing something about it’).

One participant reflected on his experiences of loneliness, and related feelings of depression, and his friend's encouragement to seek help:

I did speak to him [male friend] on the phone and he said, “[name], you need some help. You should go to the doctor because you've got a form of depression.” He said, “I know somebody else who was similar.” But I didn't go to the doctor. I should have done, I realise now. The period when I did feel quite lonely, I felt as though I was on my own and it's not a very nice place to be. [M40, 69, heterosexual]

M40’s comments suggest a state of social inertia that, while experienced as overwhelming, is temporary and situational; this does not impede him from discussing loneliness with a friend. Others emphasised the need to ‘put up with it’, as expressed by one man who did not wish to bother his adult children:

They know how I feel. “What can we do? You’ve only got to ask, and we’ll do it?” I can’t bother them. They’re very busy themselves, one way or another. You just put up with it. You just sit here and think. … You look at the clock and you think, “Oh my goodness, I’m stuck here until 11 o’clock tonight.” If I go to bed early, I can’t sleep [M104, 92, heterosexual]

Two men talked about not being able to distract themselves from loneliness and not knowing how to cope. This does not mean they were completely immobilised; they could identify some strategies for alleviating loneliness, for example telephoning friends during evenings. But getting in touch with others was a difficult social endeavour avoided and hence the cycle of loneliness would be perpetuated:

… maybe I just sit there, and feel self‐pity for myself, rather than do anything proactive about it. And I could do proactive things, I could ring people up. I could go round to people, but I don’t ‐ I tend to sit there and say, “Oh, that person hasn’t contacted me”, or, “I haven’t seen them for ages, why haven’t they contacted me?” [M63, 67, gay]

In contrast, other men stressed the importance of ‘getting on with it’ and ‘getting out there’ as an individual impetus to break loneliness and connect with individuals and groups, despite the obstacles experienced:

First of all, I’m never that bad that I can’t get out of bed, if you know what I mean … I’m never in a state where I don’t think it’s worth getting up. If I get the feeling associated with the depression and the loneliness, then I’ll just say, “Crack out of it, get on with it and sort yourself out.” [M61, 75, heterosexual]

The emphasis on ‘sorting yourself out’ aligns with masculine discourses of independence and autonomy, whereas the former position suggests a state of social inertia that may be difficult to manoeuvre out of but is not completely immobilising of support‐seeking efforts.

### 4.3 Temporary remedies and distractions from loneliness

#### 4.3.1 Keeping busy

Part of ‘sorting yourself out’ involved keeping busy—this was mentioned by most participants as a short‐term distraction that helped to keep a lid on loneliness:

You get spasms if you know what I mean? You sit down and you get a bit depressed thinking, “What am I going to do? What can I do?” It’s hard to explain loneliness. That’s why I always like to be busy, I was always busy, because no time for thinking and worrying about things. [M14, 84, heterosexual]

We frame this strategy as temporary as men described how this practice helped distract them from feelings of loneliness and associated thoughts for brief times (for example, feeling ‘empty’ or like ‘nobody cares’). However, this did not necessarily enhance their social connections with others outside the home.

Planning the day ahead with tasks and routines was a common strategy to combatting loneliness. Identified activities within the home included cooking, watching television, listening to music or going on social media. Watching television was often discussed as a distraction from loneliness. However, a number of men noted that this (lone) activity was also, at times, a reminder of their isolation:

I tell you what I feel, one of the things about living on your own as well, like I said to you before, people come along and sit back and watch television all the time. That’s a big mistake, you must get out, and you must do something. [M96, 85, heterosexual]

A small group of men stressed how creative activities provided an important distraction from loneliness –activities included music (listening and playing), writing, photography and art. For some, engagement with other media such as music helped them to adjust to, or ‘embrace’, loneliness:

There are two or three pieces of music that I play when I get into that mood, because they express that mood, and they take me into it, rather than trying to pull me away from it… And I think if I tried to run away from the feeling, the feeling would just pursue me. Whereas, if I go in and embrace it, then it passes [M8, 67, heterosexual]

The above comments indicate music as an important outlet for finding some peace with solitude and as a way of alleviating low moods associated with loneliness.

#### 4.3.2 Getting out of the home

Given that participants lived alone, it is unsurprising that the home features heavily within their reflections on tackling loneliness. In some instances, men spoke of their home as an environment of safety and security and as a symbolic space that signified long‐standing social and intimate bonds to significant others (both children and deceased spouses). In contrast, it was acknowledged by a number of participants that alleviating loneliness required getting out of the home:

So I basically don't stay in. I'm out every day. I don't like to stay in within four walls, I like to have something in my diary which I'm actually going to go somewhere or I'm going to do something specific. [M40, 69, heterosexual]

Participants emphasised the importance of routine social engagement with others, whether through mobile texting, telephoning or using social media (Facebook being most frequently named) or through planning social events and gatherings with others (for example, arranging a meet up over coffee).

Leaving the home was not always feasible for those with physical disabilities. Four men elaborated on their health conditions and disabilities that restricted their everyday mobility and opportunities to socialise with others in person. One man living in a rural area cited limited mobility and partial vision as barriers to routinely leaving the home and as trigger for loneliness:

Because, if in fact I was not disabled, if I was more mobile, more able, I would be getting out and doing more, meeting more people and mixing with more groups, in particular. [M93, 81, heterosexual]

#### 4.3.3 Maintaining connections and memories in the home

The connection with home as a space of memories and long‐lasting connection was highlighted by participants who were widowers (28 participants). While discussing feelings of loneliness, M104 referred to his deceased wife's picture in the centre of the mantelpiece and to other precious ornaments that he would not allow his home‐help assistant to dust:

It’s the only thing—the loneliness is the thing. I do feel very lonely. I talk to madame there very often [points to photo]. [M104, 92, heterosexual]

Another man frequently spoke to his deceased partner or read him an interesting extract from his book. Continuing to live in the home they had shared was a source of comfort:

I find loneliness quite an extraordinary thing. I think because I live in this house and we’ve lived here for all those years, I feel that if [partner’s name] is anywhere he is going to be here. I think that allays some of the loneliness really. [M75, 75, gay]

Within these accounts, the home is represented as a symbolic space of familiarity and historical (and ongoing) connection with significant others that bring some reprieve from feelings of loneliness.

## 5 DISCUSSION

While older people's practices for coping with loneliness at an individual and collective level have been examined in the literature, less attention has been given to older men's experiences, particularly those living alone, and the ways in which masculinity compounds coping practices. As highlighted in the findings, for some older men their current home is a symbolic space for sustaining intimate connections with deceased partners. According to Milligan (2009) for many older people the home represents a haven or protected space, a site of identity, and familiarity—in our findings, the home is a site of identity through ongoing connection to deceased partners and spouses. Morgan and Burholt (2020) argue that loss and loneliness are forms of biographical disruption. In our findings, widowed men look to biographical connections through shared histories with partners as a means of adjusting to and coping with loneliness. Returning to Schoenmakers et al.’s (2015) typology for coping, this practice is closer to a form of emotional regulation—seeking to adjust to life alone while sustaining bonds to significant others helps some men to cope with loneliness. What remains unexplored is how sustainable this strategy is over time and whether other coping practices, such as improving existing relationships, need to be deployed simultaneously.

In addition to bereavement, there were other life‐transitions interpreted by participants as central to their experiences of loneliness, including changes in relationship status and extended periods of illness and poor health resulting in declines in mobility and physical and mental functioning. The prominence of these life‐changes as precipitating factors to loneliness has been discussed in the literature (Victor, 2013; Victor et al., 2000, 2005). These life‐changes also impact older men's agency and capacity to address loneliness. In our findings disabled older men or men living with chronic health conditions experienced additional restrictions in accessing social activities outside of the home. This limits the scope for more problem‐based approaches to coping, for example accessing group‐based activities. Digital technologies may play an important role in overcoming some of these barriers (Sarwar Shah et al., 2019). Social media and email communication was one prominent strategy used by men in our study for maintaining existing social connections, rather than developing new connections.

For some men in our study who are living alone the home represents a socially restricted environment. Related to this, some men point to the sense of social inertia that inhibits them from coping with loneliness in more socially active ways, for example by looking to existing and new social connections outside of the home. Schoenmakers et al. (2015) suggest that older adults internalise beliefs about old age being associated with loneliness and hence normalise loneliness as part of their lifeworld. This partly resonates with men's experiences of social inertia as they experience a deeper sense of resignation to loneliness as part of their lifeworld—more in line with discussions of existential loneliness as a state of reflection on one's sense of estrangement and separation from the social world (Bolmsjö et al., 2018). However, this is not the case across all participants’ accounts as others emphasised loneliness as a trigger to ‘get out and do something’, indicating more assertive attempts to adjust with and cope with loneliness.

Hegemonic masculine practices endorse independence, autonomy and self‐reliance and in old age, men may attempt to retain such elements of masculinity. As Smith et al. (2007) note acting independently is both part of a discourse of masculinity and successful ageing where older men are often encouraged that to maintain independence is to ‘successfully’ age. Bennett (2007) proposes that older men negotiate emotional distress in ways that attempt to preserve masculinity with language which expresses control, rationality, responsibility and success. Aspects of this can be seen in our findings through the ways in which older men reconstruct masculinity in their descriptions of distress and loneliness and in their talk of keeping busy. Emphasising the importance of keeping busy aligns with notions of self‐reliance and independence relayed by older men across other studies (Tannenbaum & Frank, 2011). Evident within our findings are ways in which gendered expectations attached to male roles such as fatherhood, accompanied by the social stigma fastened to loneliness, thwart men's attempts to speak to others about feelings of loneliness and distress. For some older men discussing emotionally distressing problems with others, coupled with the shame attached to loneliness, can compromise expected attributes of stoicism, independence and self‐sufficiency associated with hegemonic masculinities (Tannenbaum & Frank, 2011). However, men in our study also attempted to preserve masculinity by seeking out social engagement in a way that maintained autonomy over decision making.

In recognising the differing ways in which hegemonic masculine expectations shape older men's responses to loneliness, we do not seek to elevate one response above another or suggest these are mutually exclusive. The social inertia associated with loneliness may also generate periods of self‐reflection on self, identity, and the perceived value of current social connections. These self‐reflections provide valuable points to re‐evaluate social relationships following experiences of loss or significant life change (Morgan & Burholt, 2020). The second position we describe of self‐reliance may lead to temporary improvements in levels of social engagement outside the home but equally may inhibit men from engaging in deeper reflection on how loneliness impinges on their sense of self and self‐worth.

The findings reiterate the importance of targeted individual support for older men, particularly for those feeling frozen in loneliness, alongside group‐based interventions. Group interventions may not be accessible or a realistic pursuit for many older men struggling with life‐transitions, and grief and losses associated with loneliness, at least not on a short‐term basis. But the potential social benefits from participating in group‐based programmes should not be ruled out (Cattan et al., 2005; Haggan et al., 2014); it may be that these are more a longer‐term goal for men who initially require more individualised support. Group facilitators need to be prepared to mediate potential interpersonal clashes arising between men experiencing social inertia connected to loneliness and other men advocating the imperative to rapidly address this. Men's sheds are one such peer support model where this tension may unfold. Mackenzie et al. (2017) found that among Men's Sheds participants, hegemonic norms, such as the importance of work, self‐reliance and autonomy, were initially noted but also present were discussions counter to these norms that reflected flexible masculine practices. Once through the door, the dynamic of masculinity can expand, and less traditional versions of masculinity and engagement with other men become normalised. This highlights how such groups can facilitate a space for fluidity concerning conceptualisations of multiple masculinities among male members.

There are some limitations to this study. Men were recruited through self‐selection methods from English regions (Southwest, West and Midlands regions); as such the findings have limited generalisability. Recruitment sources relied on established groups and community‐based interventions for older men and targeted older people more broadly. This limited the scope to recruit men experiencing high levels of social isolation and who were disengaged from groups and services. However, this does not diminish the value of participants’ accounts of loneliness. The sample represents the views and experiences of mostly White men and therefore we cannot speak to the ways in which racial inequalities and different ethnic identities intersect with men's experiences of masculinities, loneliness and social relations.

More longitudinal research is needed on how older men respond to and cope with loneliness to capture the experiences of moving in and out of loneliness. This is important in the context of health and social care as older men with increasing care and support needs experience changes in their living circumstances and levels of dependency on others and may experience transitions from community‐based dwellings to supported accommodation or care settings.

## CONFLICT OF INTEREST

No conflicts of interest to be declared.
